# Variant discovery in the sheep milk transcriptome using RNA sequencing

**DOI:** 10.1186/s12864-017-3581-1

**Published:** 2017-02-15

**Authors:** Aroa Suárez-Vega, Beatriz Gutiérrez-Gil, Christophe Klopp, Gwenola Tosser-Klopp, Juan José Arranz

**Affiliations:** 1 Departamento de Producción Animal, Facultad de Veterinaria, Universidad de León, Campus de Vegazana s/n, León, 24071 Spain; 2 INRA, Plateforme bioinformatique Toulouse Midi-Pyrénées, UR875 Biométrie et Intelligence Artificielle, BP 52627, 31326 Castanet-Tolosan Cedex, France; 3 GenPhySE, Université de Toulouse, INRA, INPT, ENVT, Castanet, Tolosan France

**Keywords:** Dairy Sheep, Milk Somatic Cells, RNA-Seq, Transcriptome Variants

## Abstract

**Background:**

The identification of genetic variation underlying desired phenotypes is one of the main challenges of current livestock genetic research. High-throughput transcriptome sequencing (RNA-Seq) offers new opportunities for the detection of transcriptome variants (SNPs and short indels) in different tissues and species. In this study, we used RNA-Seq on Milk Sheep Somatic Cells (MSCs) with the goal of characterizing the genetic variation within the coding regions of the milk transcriptome in Churra and Assaf sheep, two common dairy sheep breeds farmed in Spain.

**Results:**

A total of 216,637 variants were detected in the MSCs transcriptome of the eight ewes analyzed. Among them, a total of 57,795 variants were detected in the regions harboring Quantitative Trait Loci (QTL) for milk yield, protein percentage and fat percentage, of which 21.44% were novel variants. Among the total variants detected, 561 (2.52%) and 1,649 (7.42%) were predicted to produce high or moderate impact changes in the corresponding transcriptional unit, respectively. In the functional enrichment analysis of the genes positioned within selected QTL regions harboring novel relevant functional variants (high and moderate impact), the KEGG pathway with the highest enrichment was “protein processing in endoplasmic reticulum”. Additionally, a total of 504 and 1,063 variants were identified in the genes encoding principal milk proteins and molecules involved in the lipid metabolism, respectively. Of these variants, 20 mutations were found to have putative relevant effects on the encoded proteins.

**Conclusions:**

We present herein the first transcriptomic approach aimed at identifying genetic variants of the genes expressed in the lactating mammary gland of sheep. Through the transcriptome analysis of variability within regions harboring QTL for milk yield, protein percentage and fat percentage, we have found several pathways and genes that harbor mutations that could affect dairy production traits. Moreover, remarkable variants were also found in candidate genes coding for major milk proteins and proteins related to milk fat metabolism. Several of the SNPs found in this study could be included as suitable markers in genotyping platforms or custom SNP arrays to perform association analyses in commercial populations and apply genomic selection protocols in the dairy production industry.

**Electronic supplementary material:**

The online version of this article (doi:10.1186/s12864-017-3581-1) contains supplementary material, which is available to authorized users.

## Background

The identification of genetic variation underlying desired phenotypes is one of the main challenges in current dairy genetic research. The higher content of sheep milk in total solids when compared to cow and goat milk favors its greater aptitude for cheese production [1]. Therefore, genetic variation within genes that influence the total solid content of milk is of crucial interest in dairy sheep breeding because this variability could be linked to milk composition, milk quality and cheese production.

Over the years, several studies on polymorphisms in ovine major milk proteins (caseins and whey proteins) have appeared due to the potential association of these polymorphisms with milk yield, milk composition and milk technological aspects [1–4]. Additionally, as the majority of dairy sheep traits are complex, research on dairy Quantitative Trait Loci (QTL) mapping has also been widely performed. To date, 1,336 sheep QTL influencing 212 different traits have been reported in a total of 119 publications (http://www.animalgenome.org/cgi-bin/QTLdb/index; accessed at 24 November 2016) [5]. In relation to milk traits, 242 QTL have been reported [5]. However, the traditional methodology used for QTL mapping with genome-wide sparse microsatellite markers or with low/middle density Single Nucleotide Polymorphism (SNP) genotyping platforms makes it difficult to identify the true causal mutations underlying these complex traits.

Over the last few years, the constant improvement of high-throughput sequencing platforms and the availability of genome sequencing data have facilitated the detection of a substantial number of genetic variants in livestock [6, 7]. The identification of this genomic variation is crucial to the rapid identification of mutations that compromise animal health and productivity but also to build a database of polymorphisms that could be used as molecular markers for more accurate genomic predictions and genome-wide association studies [6].

High-throughput transcriptome sequencing technology (RNA-Seq) has been developed to identify and quantify gene expression in different tissues [8, 9]. Moreover, RNA-Seq also offers new opportunities for the efficient detection of transcriptome variants (SNPs and short indels) in different tissues and species [10, 11]. In this way, when compared to whole genome sequencing, RNA-Seq offers a cheaper alternative to identifying variation and, possibly, discovering the causal mutations underlying the analyzed phenotypes [12, 13].

In this study, we used RNA-Seq on Milk Sheep Somatic Cells (MSCs) with the goal of characterizing the genetic variation in the coding regions of the milk transcriptome in two dairy sheep breeds, Churra and Assaf, that are commonly farmed in Spain. In addition to the general characterization of variations in the sheep milk transcriptome, we focused our analysis on the detection of variability within the coding regions harboring QTL for milk yield, fat percentage and protein percentage and in the genes codifying for major milk proteins and enzymes related to milk fat metabolism. Thus, this analysis has allowed for the discovery of functionally relevant variants within genes related to dairy production traits that could be exploited by dairy sheep breeding programs after further research confirms the possible associations with phenotypes of interest.

## Results and discussion

### Sequencing and mapping

Milk samples from eight ewes (four Churra and four Assaf) were collected at different lactation time points (days 10, 50, 120 and 150 after lambing). Based on the quality score of the RNA (RIN > 7), we sequenced the MSCs transcriptome from eight animals on days 10, 50 and 150 of lactation and from six animals on day 120 of lactation. A total of 1,116 million paired-end reads was obtained from the transcriptome sequencing of the 30 milk samples analyzed. An alignment of the reads to the *Ovis aries* Oar_v3.1 genome yielded a mean of 88.10% of the reads per RNA-Seq sample that aligned to unique locations in the ovine genome. After merging the replicates from the same animal at the different sampling time-points and marking the duplicates on the resulting merged bam files, we found that an average of 119.33 million non-duplicated paired-end reads per animal mapped to the Oarv3.1 genome assembly. General RNA-Seq metrics obtained with the RSeQC software [14] that consider the annotation bed file of the reference sheep genome are summarized in Table 1. In our dataset of the sheep MSCs transcriptome, an average of 120.47 million tags per animal were defined. The term “tag” accounted for the number of times one read is spliced. The RSeQC program assigned an average of 110.08 million tags per merged sample to the annotated sheep genome regions. Therefore, approximately 10.39 million tags were not assigned to annotated regions, suggesting that approximately 10 million tags per sample mapped to intergenic regions. The comparative analysis performed in a previous study of the assembled transcripts of this RNA-Seq dataset with the ovine genome assembly Oar_v3.1 revealed that up to the 62% of the transcripts detected in the MSCs genome were intergenic [15]. These results reflect the incompleteness of the current annotation of the sheep transcriptome and presume the presence of non-annotated transcripts that could codify for novel proteins or constitute functional noncoding RNAs, like long noncoding RNAs (lncRNAs), microRNAs (miRNAs), short interfering RNAs (siRNAs), Piwi-interacting RNAs (piRNAs) or small nucleolar RNAs (snoRNAs). In the human genome the transcriptome functional non-coding elements have been estimated to constitute up to 98% of transcripts [16]. The identification of these functional elements in animals is one of the goals of the Functional Annotation of Animal Genomes (FAANG) project [17].

**Table 1:** Summary of sequencing results according to the annotation performed in this study of the MSC transcriptome based on the sheep genome reference Oar_v3.1

Total Reads (paired end)			119325116
Total Tags			120473958
Total Assigned			110083502
Group	Total_bases	Tag_count	Tags/kb
CDS^a^_Exons	32776750	65846229.88	2008.93
5′UTR^b^_Exons	3479917	960588.13	276.04
3′UTR^c^_Exons	8651433	4457991.13	515.29
Introns	803999021	13554137.88	16.86
TSS^d^_up_1kb	21995006	933617.50	42.45
TSS_up_5kb	101300701	2521024.00	24.89
TSS_up_10kb	187280303	3117103.63	16.64
TES^e^_down_1kb	21770670	10545653.88	484.40
TES_down_5kb	96011366	21156069.50	220.35
TES_down_10kb	173072739	22147451.25	127.97

^a^ *CDS* Coding DNA sequence; ^b^ *5′UTR* leader untranslated sequence; ^c^ *3′UTR* trailer untranslated sequence; ^d^ *TSS* Transcription Start Site; ^e^ *TES* Transcription End Site

By focusing on assigned tags, as could be expected, the vast majority of tags mapped to coding genome regions. Specifically, we found an average of 65.85 million tags per animal, or 2008.93 tags/kb that mapped to CDSs (Table 1).

### Variant detection and functional annotation

A total of 216,637 variants were detected in the MSCs transcriptome of the eight ewes analyzed after the variants were filtered (Table 2; Additional file 1). Of these variants, approximately the 78% were previously annotated in dbSNP (version 143). Among the total variants identified, 197,948 were SNPs and 18,689 were indels. The transition to transversion (Ts/Tv) ratio was 2.4, which was slightly higher than the 2.0-2.2 genome-wide Ts/Tv ratio reported in relation to human whole-genome sequence data [18]. However, this ratio is generally higher in exomes due to the increased presence of methylated cytosine in CpG dinucleotides in exonic regions [19].

**Table 2:** Summary statistics of the identified variants

Fields		Counts SnpEff	Counts VEP
Variants processed		216637	212742
	SNPs	197948	195503
	Insertions	8603	7233
	Deletions	10086	9032
Effects by impact			
	HIGH	2128	1891
	MODERATE	22440	22385
	LOW	43986	43667
	MODIFIER	312170	232768
Effects by type			
	3_prime_UTR	12940	12950
	5_prime_UTR	1819	1824
	downstream_gene	113225	113207
	frameshift	1162	1096
	inframe_deletion	168	314
	inframe_insertion	127	229
	intergenic_region	96639	16991
	intron	59198	58408
	missense	21841	21824
	non_coding_exon	2002	1993
	non_coding_transcript	10	9492
	splice_acceptor	525	332
	splice_donor	594	371
	splice_region	2353	2187
	start_lost	16	28
	stop_gained	119	112
	stop_lost	28	30
	stop_retained	26	31
	synonymous	43003	43004
	upstream_gene	27952	27948

**Fig. 1:**
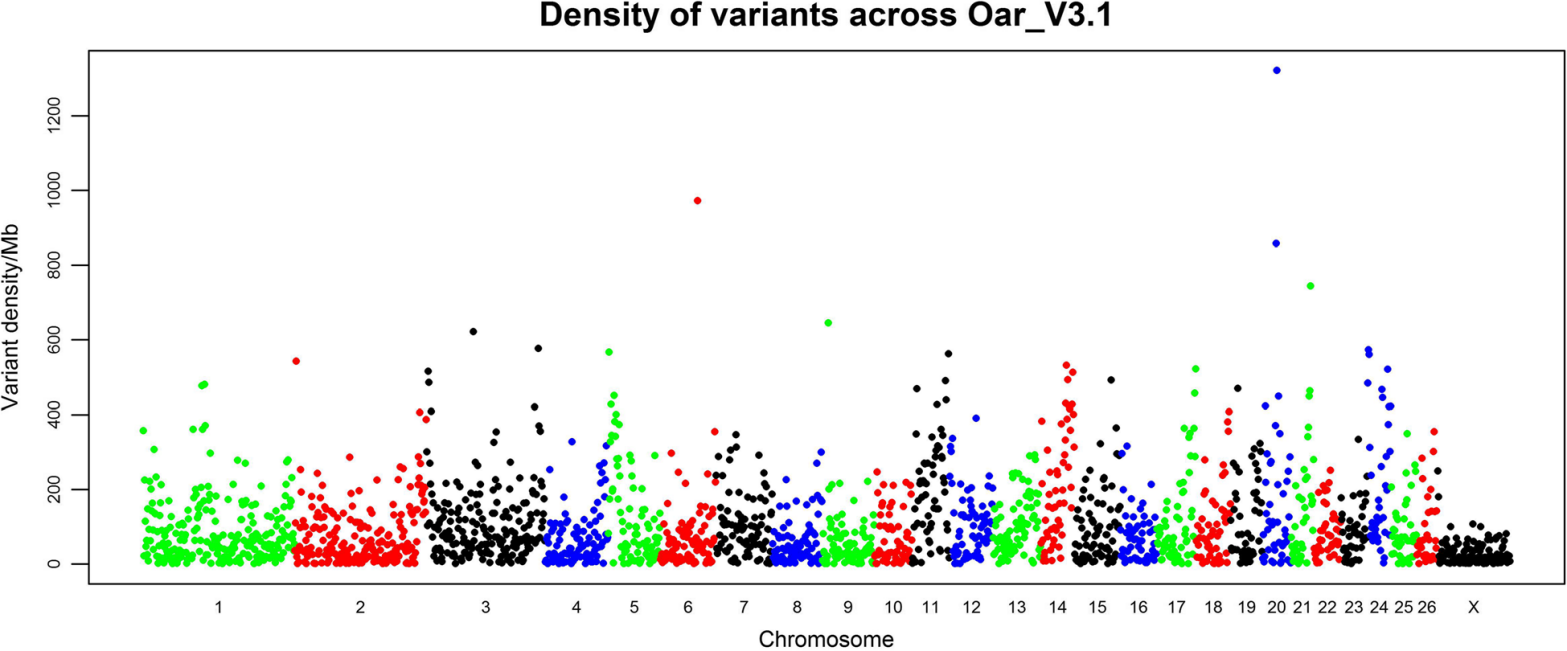
Genome-wide variant densities. Manhattan plot showing the variant density (number of SNPs per Mb) on the Y-axis and the positions of the genome across the 26 ovine autosomes and the X chromosome on the X-axis

Considering SNPs and Indels, the variant density across the genome (Fig. 1) showed a more or less uniform distribution, with three regions showing a high density of variants that should be noted (more than 800 variants/Mb). Two of these regions with high densities of variants were located on chromosome 20 (OAR20) at OAR20:26–27 Mb and OAR20:27–28 Mb, with 858 and 1321 variants/Mb, respectively. The Major Histocompatibility Complex (MHC) of sheep is located in a region of chromosome 20 [20] that corresponds to the 2 Mb region with high variability detected in this study. This region on OAR20 was also identified to harbor a putative QTL for milk yield-related traits [21]. The other region with a high number of variants (972 variants/Mb) is located on OAR6 (OAR6:85–86 Mb) and is related to the genomic location of ovine genes coding for the milk caseins (OAR6: 85,087,000-85,318,000). The large number of variants positioned in this region could be due to the high transcription levels of caseins in the lactating mammary gland. The high transcription rate of the casein cluster region, with an average of 3.48 million of tags per kb of exon, refers to the transcription of both exons and the surrounding intronic regions. Hence, it is remarkable that a very high number of tags per kb of intron was found in the casein cluster region (7011.22 tags per kb of intron) when compared with the average across the whole sheep genome (16.86 tags per kb of intron). Previous RNA-Seq analysis suggest that the pattern of the intronic sequence read coverage in RNA-Seq could be explained by an inefficient poly(A)^+^ purification [22], the presence of intronic reads flanked by poly(A)^+^ stretches [23] or by transcripts undertaking splicing after polyadenylation [23].

The annotation analyses performed with SnpEff [24] and Variant Effect Predictor (VEP) [25] are summarized in Table 2. The number of variants processed with SnpEff was higher (216,637) when compared to the variants processed with the VEP software (212,742) because SnpEff performs the annotation of the variants present in the whole domestic sheep genome (Oar_v3.1), chromosomes and scaffolds, whereas VEP only annotates variants within ovine chromosomes. Variants were assigned to four types of biological impact based on the significance of the effect of the variant: high (e.g., frame shift, stop gain/loss, start loss, etc.); moderate (e.g., nonsynonymous coding changes, codon insertion/deletion, etc.); low (e.g., synonymous changes etc.); or modifier (used for terms with hard-to-predict effects and markers) (Table 2). The number of functional effects assigned was larger than the number of loci because the categories were not mutually exclusive. Among the total number of effects detected, the vast majority of the variants were predicted to have modifier impacts by both software programs (312,170 with SnpEff and 232,768 with VEP) (Table 2). This is because most of the variants detected were located in downstream gene regions (Table 2). Among the distribution of the variants by type of effect, the results of the two annotation tools were generally consistent (Table 2). Only two non-coding categories show marked discrepancies as follows: the variants annotated as intergenic regions and the variants annotated as non-coding transcript variants (Table 2). A higher number of variants were found by SnpEff than by VEP in intergenic regions (96,639 and 16,991, respectively), which could be due to the different performances of the annotation algorithms. The VEP software found a greater number of non-coding transcript variants than SnpEff (9,492 and 10 variants, respectively) because VEP annotates regulatory region variants without providing additional datasets to the software [25].

Among the results described in Table 2, it is remarkable the large proportion of variants identified within non-coding regions (e.g. downstream, intergenic, intronic variants) which could indicate the presence of variants in unannotated exons and/or noncoding but functionally transcribed genomic regions. As we have pointed above, the 62% of the transcripts detected within the ovine MSCs transcriptome were intergenic and moreover, the 11% were classified as potentially novel isoforms [15]. Therefore, the detection of variants out of known protein coding regions can be expected. Furthermore, these results agree with the results found in previous studies in cattle and human [26, 27]. However, further research needs to be done in the identification of transcriptome functional elements in livestock genomes to elucidate the potential role of the variants detected within no-coding regions.

### Variants in QTL regions

A total of 57,795 variants were detected within the selected regions harboring QTL for milk yield, protein percentage and fat percentage. Among them, 78.56% were mutations already described in SNPdb (version 143). Most QTL in dairy sheep have been mapped with low-density maps, resulting in the detection of the significant effect within large confidence intervals. Hence, the high amount of variants detected in this work within ovine QTL for dairy traits could be related to the low mapping resolution of many of the previously identified QTL effects.

**Fig. 2:**
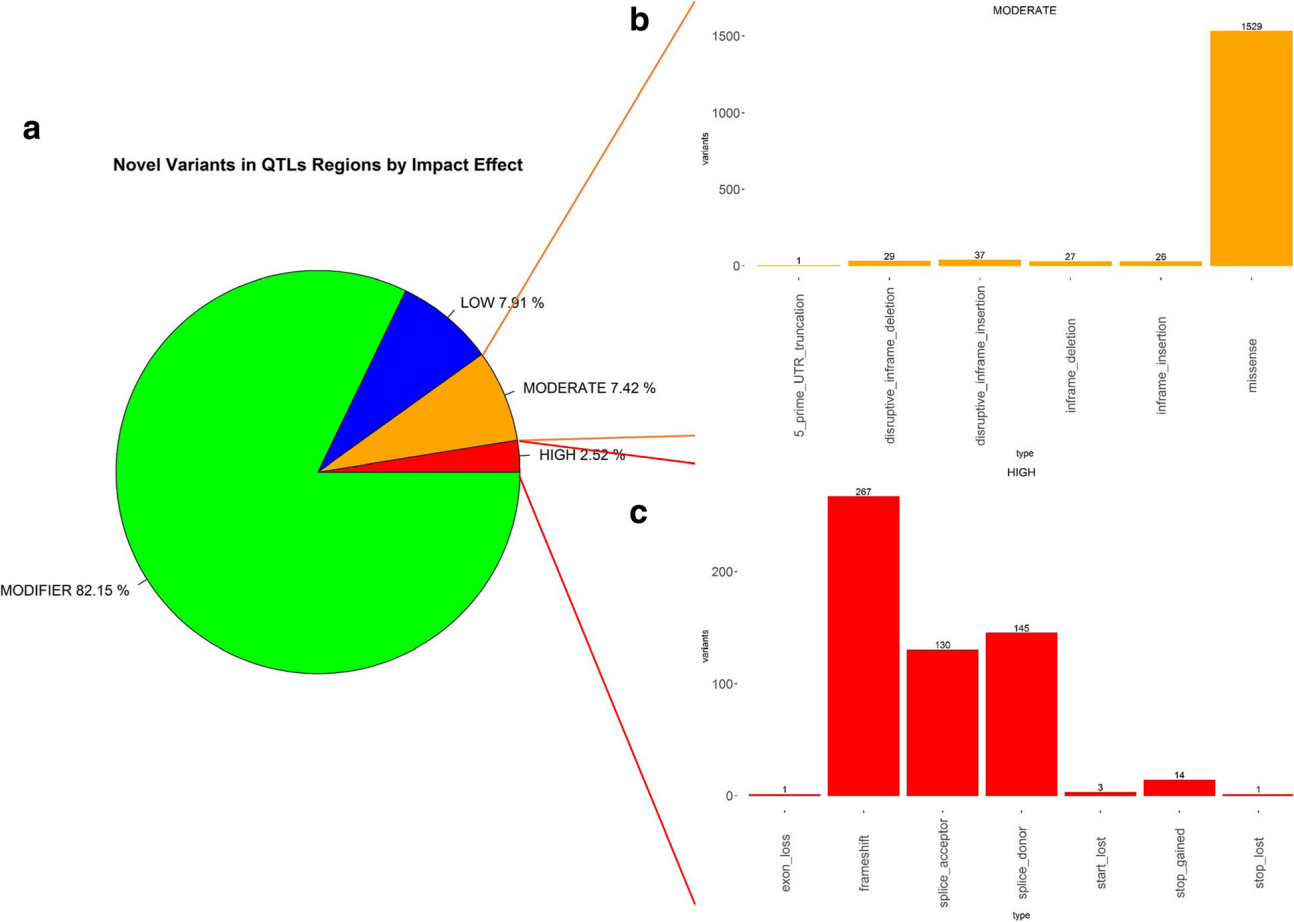
Functional characterization established by SnpEff and VEP software for the novel variants identified in this study within the QTL previously reported for milk yield, milk protein percentage and milk fat percentage. **a** Distribution of the novel variants by impact; **b** Distribution of moderate impact novel variants within QTL regions by functional effect; **c** Distribution of high impact novel variants within QTL regions by functional effect

Due to the large number of total variants found, we focused our further exploratory study on the novel variants detected. Among the 12,389 novel variants identified within QTL regions, 9,118 were SNPs, 2,161 were insertions and 1,110 were deletions. Approximately 82.15% of the identified novel variants were considered sequence modifiers; the remaining (~17,85%) were inferred to produce high impact (2.52%), moderate impact (7.42%) or low impact (7.91%) changes in the corresponding transcriptional unit (Fig. 2).

Considering that the variants found within QTL regions may have been a consequence of selective pressures related to dairy production traits, we performed a functional enrichment analysis of the genes containing the variants with high and moderate functional impacts. For this analysis, we considered the variants that were classified as high and moderate impact variants (Fig. 2) by the two annotation software programs used, SnpEff [24] and VEP [25]. However, based on the large number of moderate missense variants identified by both programs (Fig. 2), we performed additional filtering to consider only the missense mutations predicted to be deleterious by SIFT [28], an external tool implemented in the VEP software that predicts the effects of an amino acid substitution on protein function. Hence, after discarding those variants predicted to be tolerated, a final total of 371 unique genes containing relevant functional variants (Additional file 2) were used to perform a functional enrichment analysis using the WEB-based Gene SeT AnaLysis Toolkit (WebGestalt) [29]. These genes were categorized by 14 enriched KEGG (Kyoto Encyclopedia of Genes and Genomes) pathway terms (*p*_*ad*j_ < 0.05) (Additional file 3). The highest enriched KEGG pathway was “protein processing in endoplasmic reticulum” with a *p*_*ad*j_ of 2.60e-05. Metabolic processes in endoplasmic reticulum (ER) are associated with the synthesis and folding of membrane and secretory proteins as well as lipid synthesis. Under certain stress conditions (such as high levels of carbon-based molecules, free fatty acids, cytokines, and hypoxia), the accumulation of unfolded/misfolded proteins activates the ER stress signaling response [30, 31]. The mammary gland faces high metabolic stress during lactation due to the elevated rates of protein and fat synthesis. In our study, the majority of the genes with relevant functional variants enriched in the KEGG pathway “protein processing in ER” were related to the ER stress response (*CAPN2*, *HSP90B1*, *PLAA*, *DERL2*, *DNAJB2*, *VCP*, *UBQLN1*, *SSR1*). Mutations in these genes could be related to a different response of the overloaded ER in mutated animals during lactation, suggesting that these mutations could be a consequence of selective pressure for milk production traits. The high and moderate impact variants found in these genes and the animal genotypes for these variants are summarized in the additional information (Additional file 4).

Among the remaining enriched KEGG pathways (*p*_*ad*j_ < 0.005) found in this analysis (Additional file 3), “Jak-STAT signaling pathway”, “RNA transport” and “Fatty acid elongation” should be highlighted due to the putative influence of the genes within these pathways in milk yield or milk protein and fat content (see relevant variants and associated genes in Additional file 4). The Jak-STAT signaling pathway is directly implicated in milk protein expression by the mammary gland during lactation [32, 33]. Among the variants found in the genes within this pathway, the variant found in the *signal transducer and activator of transcription 4 (STAT4)* gene is noteworthy because variants in the orthologous bovine gene have been significantly associated with milk yield and protein percentage [34, 35].

In the “RNA transport” pathway, it is worthwhile to highlight variants within the *EIF4G3*, *EIF3I*, and *EIF3D* genes. These three genes code for the eukaryotic translation initiation factors 4 Gamma 3, 3 Subunit I and 3 Subunit D, respectively. The binding of eIF4G to eIF3 is regulated by insulin via the association of mTOR with eIF3, which causes the initiation of translation in the mTOR signaling pathway [36, 37]. This pathway is implicated in the positive control of protein synthesis, and studies in ruminants have highlighted the crucial role of the mTOR signaling pathway in the regulation of milk protein synthesis [38].

The following two genes were enriched in the “Fatty acid elongation in mitochondria” KEGG pathway: *PPT2* and *ACAA2. PPT2* is located within the ovine MHC region and encodes a member of the palmitoyl-protein thioesterase family, which has significant thioesterase activity against lipids with chain lengths of 10 or fewer carbons and 18 or more carbons [39]. The *ACAA2* gene codes for the acetyl-CoA acyltransferase 2, a protein involved in lipid metabolism that catabolizes the last step in fatty acid β-oxidation. In Chios sheep, a single nucleotide polymorphism in *ACAA2* was identified and associated with the milk yield phenotype [40].

### Variants in sheep-cheese candidate genes

#### Variants in genes related to milk protein content

Variability related to milk protein content was evaluated in the genes codifying for major milk proteins, i.e., within the genes encoding caseins (*casein α-S1* (*CSN1S1*), *casein α-S2* (*CSN1S2*), *casein β* (*CSN2*), and *casein κ* (*CSN3*)) and whey proteins (*α-lactalbumin* (*LALBA*) and *β-lactoglobulin* (*PAEP*)). After variant filtration a total of 504 variants were identified within these genes. Among these variants, 80 (15.9%) variants were novel, and 424 (84.1%) variants were previously annotated in SNPdb (version 143). Most of the detected variants in the major milk protein genes (452) were single nucleotide polymorphisms (SNPs). There were also 29 deletions and 23 insertions.

A high number of the variants found in the genes codifying for major milk proteins were positioned in introns (482). The large number of tags mapped to introns within the casein cluster, which was pointed above, together with the higher variability generally expected in non-coding regions may explain the high level of genetic variation identified in this region.

Among the variants detected in the coding regions by both software programs (SnpEff and VEP), we found one splice donor variant, which was classified as a high impact effect mutation, and ten missense variants. These mutations found within protein genes are summarized in Table 3. The splice donor variant found in the *CSN1S2* gene is a novel variant that was detected in the two studied breeds (allele frequency of 0.625). This variant affects a putative splice donor site at the third intron of the *CSN1S2* gene (*GCA_000298735.1:6:85186875:G:A*). Thus, this SNP could cause intron retention resulting in a novel isoform of *CSN1S2*, which should be confirmed by further research.

**Table 3:** Functionally relevant variants in genes codifying for major milk proteins

Variant ^a^	Gene	Allele Freq		Effect	AA
		Assaf	Churra		
*rs600923112*	*PAEP*	0.25	0.5	Missense-Deleterious	*p.Gln167Leu*
*rs600923112*	*PAEP*	0.375	0	Missense-Deleterious	*p.Gln167Arg*
*rs430610497*	*PAEP*	0.375	0.5	Missense-Tolerated	*p.His36Tyr*
*rs403176291*	*LALBA*	0.125	0.5	Missense-Deleterious	*p.Val27Ala*
*rs420959261*	*CSN1S1*	0.38	0.75	Missense-Tolerated	*p.Thr209Ile*
*rs416941267*	*CSN2*	0.625	0.25	Missense-Tolerated	*p.Leu212Ile*
*rs430298704*	*CSN2*	0	0.125	Missense-Tolerated	*p.Met199Val*
*GCA_000298735.1:6:85186875:G:A*	*CSN1S2*	0.625	0.625	Splice donor	
*rs430397133*	*CSN1S2*	0	0.125	Missense-Deleterious	*p.Asp90Tyr*
*rs424657035*	*CSN1S2*	0	0.25	Missense-Tolerated	*p.Ile120Val*
*rs399378277*	*CSN1S2*	0.125	0.75	Missense-Tolerated	*p.Arg176His*

^a^ For described variants *rs* identifier is indicated and novel variants are described with the unique ID “*INSDC Genome accession:CHROM:POS:REF:ALT*”.

Missense variants in the ovine casein genes, which lead to amino acid changes in the protein products, comprise a group of SNPs that are of particular interest because some of these variants have been demonstrated to influence the composition and/or technological properties of milk (reviewed by Moioli et al. [41]). Among the missense variants detected in this study (Table 3), one was in *CSN1S1*, two were in *CSN2* and three were in *CSN1S2*; no missense variants were found in *CSN3*. This result agrees with the fact that *CSN3* is considered to be monomorphic in sheep [1]. Missense variants detected in the *CSN1S2* gene are relevant due to their relationships with known protein alleles. The deleterious variant *rs430397133* was detected in the *CSN1S2* gene in one heterozygous Churra ewe (allele frequency of 0.125). The same animal was heterozygous for the other two missense variants found in *CSN1S2*, named *rs424657035* and *rs399378277*, which were predicted to be tolerated. The mature protein of the known CSN1S2*B’ variant harbors these three missense mutations [42]. The deleterious variant *rs430397133*, which causes the *Asp90Tyr* substitution, is responsible for the higher isoelectric point of the B protein variant that allows for its differentiation from CSN1S2*A [43]. An advantageous effect of CSN1S2*B in comparison to CSN1S2*A in terms of milk, fat and protein yield, and protein content has been reported [3]. In this study, we also found the variants responsible for αs2-CN protein alleles G (*rs424657035*) and G’ (*rs424657035* and *rs399378277*). However, at the protein level, the G and G’ alleles are hidden by the CSN1S2*A phenotype in isoelectric focusing [3].

In the *CSN1S1* gene, we found a previously described missense variant (*rs420959261*). This SNP is responsible for the *p.Thr209Ile* substitution, which differentiates the protein variant CSN1S1*C’, the supposed ancestral variant, from CSN1S1*C” [44].

Two known SNPs, *rs430298704* and *rs416941267*, were detected within the *CSN2* gene. The *rs430298704* SNP is a missense variant causing the substitution *p.Met199Val* which is classified as tolerated. This mutation causes the A and G protein alleles of β-casein. Corral et al. [45] found that in Merino sheep the GG genotype for this variant was associated with an increase in milk production, whereas the AA genotype was associated with an increase in protein and fat percentage. The *rs416941267* is a missense variant causing the amino acid exchange *p.Leu212Ile* associated to the CSN2*X protein allele described by Chessa et al. [46].

One already described missense SNP, *rs403176291*, was detected within the *LALBA* gene in both breeds. This mutation causes the amino acid change *p.Val27Ala* classified as deleterious by SIFT [28] and that has been suggested to be a Quantitative Trait Nucleotide (QTN) influencing milk protein percentage [47].

Regarding the *PAEP* (*LGB*) gene, which encodes the milk β-lactoglobulin protein, our analysis identified the missense variant (*rs430610497*) that differentiates protein alleles A and B of β-lactoglobulin [48, 49]. This mutation causes the substitution *p.Tyr36His* and was found in both breeds. A higher aptitude for cheese processing has been shown in AA ewes due to a shorter clotting time, better rate of curd firming and a higher cheese yield [2]. The C allele of β-lactoglobulin [50] was not found in this study. This rare C variant has been only found in few breeds, including Merinoland, Latxa, Carranzana, Spanish Merino, Serra da Estrela, White Merino, and Black Merino [2]. However, at position c.500 of the *PAEP* gene, we detected trialelic missense variants, *rs600923112* and *rs600923112*, which cause two amino acid substitutions in the protein (*p.Gln167Leu* and *p.Gln167Arg*, respectively)*.* The *p.Gln167Leu* amino acid change was found in the two studied breeds, whereas the *p.Gln167Arg* substitution was found only in Assaf sheep. These seem to be important mutations, as both amino acid changes are predicted to be deleterious by SIFT [28]. To our knowledge, these mutations are not related to described protein alleles in the β-lactoglobulin so further research should be conducted to elucidate their possible functional consequences.

#### Variants in genes related to milk fat content

To find variability in candidate genes related to milk fat content, we filtered the mutations positioned within a total of 17 genes (Table 4) that have been previously related to milk fat metabolism [51].

**Table 4:** Milk fat candidate genes considered in this study

Gene symbol	Description
*BTN1A1*	*Butyrophilin Subfamily 1 Member A1*
*ACACA*	*Acetyl-CoA Carboxylase Alpha*
*FABP3*	*Fatty Acid Binding Protein 3*
*CEL*	*Carboxyl Ester Lipase*
*ACSL1*	*Acyl-CoA Synthetase Long-Chain Family Member 1*
*LPL*	*Lipoprotein Lipase*
*ACSS2*	*Acyl-CoA Synthetase Short-Chain Family Member 2*
*XDH*	*Xanthine Dehydrogenase*
*GPAM*	*Glycerol-3-Phosphate Acyltransferase, Mitochondrial*
*DBI*	*Diazepam Binding Inhibitor, Acyl-CoA Binding Protein*
*VLDLR*	*Very Low Density Lipoprotein Receptor*
*DGAT1*	*Diacylglycerol O-Acyltransferase 1*
*PLIN2*	*Perilipin 2*
*SCD*	*Stearoyl-CoA Desaturase*
*LPIN1*	*Lipin 1*
*SLC27A6*	*Solute Carrier Family 27 Member 6*
*FASN*	*Fatty Acid Synthase*

We detected a total of 1,063 variants in the transcriptomic regions containing the studied genes related to lipid metabolism. The majority of the variants within these genes (953; 89.65%) were previously annotated in SNPdb (version 143). Among the variants detected, 990 were SNPs, 24 were insertions, and 49 were deletions. As these variants occurred in the genomic regions encoding caseins and whey proteins, the highest proportion of mutations were located within intronic regions (920; 86.39%).

According to the functional effects by impact found in the fat-related genes, we identified four (0.38%) variants with high impact, 27 (2.54%) with moderate impact, 100 (9.39%) with low impact and 934 (87.7%) with a modifier impact. Among the moderate variants, we found a disruptive inframe deletion and 26 missense mutations, of which four were classified as deleterious by SIFT [28]. The functionally relevant variants within genes related to mammary gland fat metabolism are indicated in Table 5.

**Table 5:** Functionally relevant variants detected in the milk fat candidate genes considered in this study

Variant^a^	Gene	Allele Freq		Effect	AA
		Assaf	Churra		
*GCA_000298735.1:2:87107748:C:A*	*PLIN2*	0.5	0.5	High-Splice donor	
*GCA_000298735.1:3:20585665:C:T*	*LPIN1*	0.125	0	Missense-Deleterious (0)	*p.Arg781Trp*
*GCA_000298735.1:3:92183603:G:T*	*XDH*	0.5	0.5	High-Splice aceptor	
*rs428221119*	*XDH*	0.25	0	Missense-Deleterious (0.02)	*p.Leu246Phe*
*rs429850918*	*XDH*	0.25	0	Missense-Deleterious (0)	*p.Arg614Trp*
*GCA_000298735.1:3:92217135:G:A*	*XDH*	0.5	0.5	High-Splice aceptor	
*GCA_000298735.1:3:92239411: CCGCCCCTCTTCCCGGGCGCCCCCATCTTCTTTTCCA:C*	*XDH*	1	1	Moderate-Inframe deletion	*p.Pro1251_Phe1262del*
*rs604791005*	*FASN*	0	0.125	Missense-deleterious-low_confidence (0.04)	*p.Gly2312Ala*
*GCA_000298735.1:26:13949071:C:T*	*ACSL1*	0.5	0.5	High-Splice donor	

^a^ For described variants *rs* identifier is indicated and novel variants are described with the unique ID “*INSDC Genome accession:CHROM:POS:REF:ALT*”

The highest number of functionally relevant variants were found in the *XDH* gene. Two splice acceptor mutations and an inframe deletion were found in both breeds (Table 5). It should be noted that the inframe deletion (*GCA_000298735.1:3:92239411:CCGCCCCTCTTCCCGGGCGCCCCCATCTTCTTTTCCA:C*) was found in homozygosis in the eight ewes analyzed, which could mean that the *XDH* sequence is not well-characterized at this genomic location. Moreover, two deleterious missense SNPs were found only in Assaf ewes (allele frequency of 0.125). *XDH* encodes the xanthine dehydrogenase, a protein implicated in milk fat globule secretion [52]. Hence, mutations in this gene could alter the mechanisms underlying lipid droplet secretion.

*PLIN2* encodes the perilipin 2/adipophilin protein. Adipophilin is reported to have a role in the packaging of triglycerides for secretion as milk lipids in the mammary gland [53]. Moreover, the absence of adipophilin has been associated with the formation of smaller intracellular fat globules [54]. The splice donor variant found within *PLIN2* (*GCA_000298735.1:2:87107748:C:A*) gene is a novel variant that was detected in both breeds (allele frequency of 0.5). This variant affects a splice donor site at the first intron of the *PLIN2* gene. Thus, this SNP could cause intron retention and a novel isoform.

A novel missense variant within the *LPIN1* gene (*GCA_000298735.1:3:20585665:C:T*), causing the amino acid substitution *p.Arg781Trp* at the protein level, and classified as deleterious by SIFT [28], was found in heterozygosis in one Assaf sheep. *LPIN1* encodes the lipin-1 protein, an enzyme implicated in triacylglycerol synthesis [32]. Additionally, a role for lipin-1 in the transcriptional regulation of other genes involved in milk lipid synthesis has been suggested in relation to the mTOR, PPARα and PPARγ regulatory pathways [55–57].

In the *FASN* gene, we detected a known missense mutation (*rs604791005*) that causes the amino acid change *p.Gly2312Ala*. This polymorphism was found in heterozygosis in one Churra ewe. *FASN* encodes a fatty acid synthase responsible for *de novo* fatty-acid biosynthesis in the mammary gland [58]. In cattle, several polymorphisms in this gene have been associated with milk fat content and fatty acid composition [59–64]. In Churra sheep, two QTL affecting capric acid and polyunsaturated fatty acid contents were mapped to the genomic region harboring the *FASN* gene [65], although the variability identified in this gene did not appear to be directly related to these QTL [65]. Therefore, the missense polymorphism described in this study should be further analyzed to assess its possible association with the QTL previously described in Churra sheep.

The splice donor variant found in the *ACSL1* gene is a novel variant that was detected in both breeds (allele frequency of 0.5). This variant (*GCA_000298735.1:26:13949071:C:T*) affects the first base of the 5′ splice donor region of the second intron of *ACSL1*, which encodes an acyl-CoA synthetase long-chain family member 1. This protein is implicated in the activation of long chain fatty acids [32].

## Conclusions

We present herein the first transcriptomic approach performed to identify the genetic variants of the lactating mammary gland in sheep. Through the transcriptome analysis of variability within regions harboring QTL for milk yield, protein percentage and fat percentage, we found several pathways and genes that could harbor mutations with relevant effects on dairy production traits. Moreover, remarkable variants were also found in candidate genes coding for major milk proteins and enzymes related to milk fat metabolism. Further research is required to estimate the allele frequencies and determine the phenotypic effects of the functionally relevant variants found through this RNA-Seq approach in commercial sheep populations. Additionally, several of the SNPs found in this study could be included as suitable markers in genotyping platforms or custom SNP-arrays to perform association analyses in commercial populations and apply genomic selection protocols in the dairy production industry.

## Methods

### Animals and sampling

For this study, a MSCs transcriptome dataset from Assaf and Spanish Churra dairy sheep breeds was used. The dataset is available in the Gene Expression Omnibus (GEO) database under the accession number GSE74825. The source of the animals and the sampling process protocol are described in detail in the related data descriptor manuscript [66]. The milk samples of eight healthy sheep (four Churra and four Assaf ewes) belonging to the commercial farm of the University of León were collected on days 10 (D10), 50 (D50), 120 (D120) and 150 (D150) after lambing. At each sampling time-point, we collected 50 ml of milk from each ewe one hour after the routine milking at 8 a.m. and ten minutes after the administration of five IUs of Oxytocin Facilpart (Syva, León, Spain). The time-point for milk collection was chosen to maximize the concentration of MSCs. Previous studies have indicated that the diurnal time point with the highest concentration of MSCs occurs one hour after milking [67]. Moreover, oxytocin was administered with the aim of stimulating its mechanical effect on myoepithelial contraction and thus the flattening of the alveolar lumen, which causes the release of residual post-milking milk containing a higher concentration of exfoliated MECs [68].

### Ethics statement

All protocols involving animals were approved by the Animal Welfare Committee of the University of Leon, Spain, following the proceedings described in Spanish and EU legislations (Law 32/2007, R.D. 1201/2005, and Council Directive 2010/63/EU).

### Library preparation and sequencing

Somatic cell separation and RNA extraction were performed as described by Suárez-Vega et al. (2016) [66]. The integrity of the RNA was assessed using an Agilent 2100 Bioanalyzer device (Agilent Technologies, Santa Clara, CA, USA). The RNA integrity value (RIN) of the samples ranged between 7.1 and 9. Paired-end libraries with fragments of 300 bp were prepared using the True-Seq RNA-Seq sample preparation Kit v2 (Illumina, San Diego, CA, USA). The fragments were sequenced on an Illumina Hi-Seq 2000 sequencer (Fasteris SA, Plan-les-Ouates, Switzerland).

### Alignment, variant identification and annotation

The read qualities of the RNA-Seq libraries were evaluated using FastQC [69]. Using the STAR aligner [70] the reads were mapped against the ovine genome assembly v.3.1. (Oar_v3.1 [71]). After the alignment, Samtools [72] was used to convert sam files to bam files and then to sort and merge the bam files from the same animal at different time-points. Metrics from the bam files were obtained with RSeQC software [14] based on the annotation bed file of the Oar_v3.1 sheep assembly obtained from the UCSC Genome Browser [73]. Then, Picard [74] was used to add read groups and mark duplicated reads on the merged bam files. SNP and Indel calling was performed using the Genome Analysis Toolkit (GATK, version 3.4.46) software package following GATK best practices [75]. To obtain high-quality variants, strict filter conditions were applied using vcffilter [76] and SnpSift [77] (Variation Quality (QUAL) >30, Mapping Quality (MQ) >40, Quality By Depth. (QD) >5, Fisher Strand (FS) <60 and a minimum Depth of coverage (DP) >5 in all the samples). The bcftools “annotate –c ID” option [72] and the ovine reference vcf file downloaded from the Ensembl database (SNPdb-version 143) were used to annotate the known variants detected in our study.

Two software programs, SnpEff [24] and Variant Effect Predictor [25], were used to predict the functional consequences of the detected variants. SnpEff allows users to define specific intervals and customize the annotation of the variants. Considering that the final aim of this study is the characterization of the transcriptome variants that may be of special interest for the dairy industry, we used SnpEff to select (i) the variants included within previously reported sheep QTL studies for milk protein percentage, milk fat percentage and milk yield [5] and (ii) the variants included within candidate genes related to milk protein and fat content. The selection of the variants included in these two types of target regions (QTL and candidate genes) was performed according to the following criteria.

#### Filtering variants in QTL regions affecting milk production traits

The coordinates of the genomic regions containing the QTL related to milk protein percentage, milk fat percentage and milk yield, based on the annotation of the SheepQTLdb [5], were downloaded from the Ensembl database [71]. This information, provided as a bed file (Additional file 5), was used by the SnpEff software (−*fi* option) to retain only the variants matching the target QTL intervals from the total number of variants identified through the GATK protocol. Due to the high number of variants detected in the selected QTL regions (57,795), those variants already described in the Ensembl database were filtered out using vcftools [78]. Among the novel variants, we selected those which were predicted by the two annotation analyses (SnpEff and VEP) to have relevant functional consequences. Thus, we retained those variants that were classified in terms of their functional consequences as “high” and “moderate” by the two different software programs. Due to the large number of variants classified as “moderate”, within the moderate missense variants, we selected those predicted to be “deleterious” by the VEP option “--sift b” [25]. This option allows the use of the SIFT tool [28] for any of the variants annotated as missense. SIFT is an algorithm that predicts whether an amino acid substitution will have a deleterious effect on the protein function [28]. Finally, we extracted the names of the genes containing these functionally relevant mutations and used them to perform a functional enrichment analysis with the Web-based Gene Set Analysis Toolkit (WebGestalt) [29].

#### Filtering variants on protein and fat candidate genes

The candidate genes selected for a detailed analysis of their genetic variability in the studied dataset included those codifying for major milk constituent proteins (*CSN1S1, CSN1S2, CSN2, CSN3, PAEP, LALBA*) and 17 genes related to mammary gland lipid metabolism (Table  4). These genes were selected based on a previous study by our research group that evaluated the gene expression of candidate milk genes in the milk sheep transcriptome that affect cheese-related traits [51]. To obtain the variants within the target genes selected for the study, we used the *–fi* option from SnpEff followed by a bed file with the coordinates of the selected genes (Additional files 6 and 7) and the *–onlyTr* option followed by a file with an ID list with the Ensembl transcripts name of the selected genes. From all the variants detected within the candidate cheese-yield genes, we focused further our analyses on those mutations that could have relevant consequences. Hence, the variants classified by the two software programs as having “high” and “moderate” functional impacts were selected.

## Additional files

Additional file 1: Title of data: Variants detected within the sheep milk transcriptome. Description of data: Worksheet providing all the variants detected within the milk somatic cells transcriptome. (XLSX 60502 kb)
Additional file 2: Title of data: Genes in QTL regions containing relevant functional variants. Description of data: Worksheet providing the list of genes within QTL regions, which contain variants with functional interest. (XLSX 15 kb)
Additional file 3: Title of data: Results of the KEGG pathway enrichment analysis with the genes in QTL regions containing relevant functional variants. Description of data: Worksheet providing the results of the KEGG pathway enrichment analysis performed with the genes containing variants with functional interest. The file provides the enriched KEGG pathways, with the *p-values* and the genes grouped within each pathway. (XLSX 15 kb)
Additional file 4: Title of data: Functionally relevant variants found in the genes in “NOD-like receptor signaling pathway”, “Protein processing in endoplasmic reticulum”, “RNA tansport” and “Fatty acid elongation in mitochondria” KEGG pathways. Description of data: Worksheet providing the description and phenotypes of the functionally relevant variants found in the genes in “NOD-like receptor signaling pathway”, “Protein processing in endoplasmic reticulum”, “RNA tansport“and “Fatty acid elongation in mitochondria” KEGG pathways. (XLSX 15 kb)
Additional file 5: Title of data: Genomic regions containing the QTL related to milk protein percentage, milk fat percentage and milk yield. Description of data: Worksheet providing the coordinates of the genomic regions containing the QTL related to milk protein percentage, milk fat percentage and milk yield, based on the annotation of the SheepQTLdb. (XLSX 12 kb)
Additional file 6: Title of data: Coordinates of the milk protein genes genomic regions. Description of data: Worksheet providing the coordinates of the genomic regions containing the milk protein genes. (XLSX 9 kb)
Additional file 7: Title of data: Coordinates of the milk fat genes genomic regions. Description of data: Worksheet providing the coordinates of the genomic regions containing the genes related to milk fat metabolism. (XLSX 10 kb)

## Abbreviations

*ACAA2*: *Acetyl-CoA Acyltransferase 2*
*ACACA*: *Acetyl-CoA Carboxylase Alpha*
*ACSL1*: *Acyl-CoA Synthetase Long-Chain Family Member 1*
*ACSS2*: *Acyl-CoA Synthetase Short-Chain Family Member 2*
ALT: Alternative Allele
*BTN1A1*: *Butyrophilin Subfamily 1 Member A1*
CDS: Coding Sequence
*CEL*: *Carboxyl Ester Lipase*
CHROM: Chromosome
*CSN1S1*: *Casein Alpha S1*
*CSN1S2*: *Casein Alpha S2*
*CSN2*: *Casein Beta*
*CSN3*: *Casein Kappa*
D10: Day 10 after lambing
D120: Day 120 after lambing
D150: Day 150 after lambing
D50: Day 50 after lambing
*DBI*: *Diazepam Binding Inhibitor, Acyl-CoA Binding Protein*
dbSNP: NCBI database of genetic variation
*DGAT1*: *Diacylglycerol O-Acyltransferase 1*
DP: Depth of Coverage
*EIF3D*: *Eukaryotic Translation Initiation Factor 3 Subunit D*
*EIF3I*: *Eukaryotic Translation Initiation Factor 3 Subunit I*
*EIF4G3*: *Eukaryotic Translation Initiation Factor 4 Gamma 3*
ER: Endoplasmic Reticulum
EU: European Union
FAANG: Functional Annotation of Animal Genomes
*FABP3*: *Fatty Acid Binding Protein 3*
*FASN*: *Fatty Acid Synthase*
FS: Fisher Strand
GATK: Genome Analysis Toolkit
GEO: Gene Expression Omnibus
*GPAM*: *Glycerol-3-Phosphate Acyltransferase, Mitochondrial*
ID: Identifier
Indels: Insertions/Deletions
INSDC Genome accession: International Nucleotide Sequence Database Collaboration Genome accesion
IUs: International Units
kb: Kilobase
KEGG: Kyoto Encyclopedia of Genes and Genomes
*LALBA*: *Lactalbumin Alpha*
lncRNAs: long noncoding RNAs
*LPIN1*: *Lipin 1*
*LPL*: *Lipoprotein Lipase*
Mb: Megabase
MECs: Milk Epithelial Cells
MHC: Major Histocompatibility Complex
miRNAs: microRNAs
MQ: Mapping Quality
MSCs: Milk Somatic Cells
mTOR: Mechanistic Target Of Rapamycin
*PAEP (LGB)*: *Progestagen Associated Endometrial Protein (Beta-lactoglobulin)*
piRNAs: Piwi-interacting RNAs
*PLIN2*: *Perilipin 2*
POS: Position in the chromosome
PPARα: Peroxisome Proliferator Activated Receptor Alpha
PPARγ: Peroxisome Proliferator Activated Receptor Gamma
*PPT2*: *Palmitoyl-Protein Thioesterase 2*
QD: Quality By Depth
QTL: Quantitative Trait Loci
QUAL: Variation Quality
REF: Reference Allele
RIN: RNA Integrity Number
RNA: Ribonucleic acid
RNA-Seq: RNA sequencing
*SCD*: *Stearoyl-CoA Desaturase*
siRNAs: short interfering RNAs
*SLC27A6*: *Solute Carrier Family 27 Member 6*
snoRNAs: small nucleolar RNAs
SNP: Single Nucleotide Polymorphism
SnpEff: Genetic variant annotation and effect prediction toolbox
*STAT4*: *Signal Transducer And Activator Of Transcription 4*
Ts/Tv: Transition to Transversion
VEP: Variant Effect Predictor
*VLDLR*: *Very Low Density Lipoprotein Receptor*
WebGestalt: WEB-based Gene SeT AnaLysis Toolkit
*XDH*: *Xanthine Dehydrogenase*
